# Neurology

**Published:** 1904-09-03

**Authors:** 


### PROGRESS IN MEDICINE AND SURGERY.

### NEUROLOGY.

(*Continued from page* 380.)

**Argyll-Robertson Pupil.**—The anatomical basis of this well-known phenomenon is the subject of a discussion by Thomas.[6] Contracted pupils in association with tabes had been pointed out long before Argyll-Robertson's papers, but he in 1869 first described this symptom, viz., "that although the retina is quite sensitive and the pupils contract during the act of accommodation, yet an alteration in the amount of light admitted to the eye does not influence the size of the pupil." This is only present in tabes and general paresis, with the exception of extremely rare focal lesions in the region of the corpora quadrigemina. The symptom was first called after its describer by Grainger Stewart in 1879-80. The solution of the problem of the site of the lesion causing the Argyll-Robertson pupil is still to be found. The anatomical paths, as yet determined, are as follows:—The sphincter and dilator muscles of the iris are composed of smooth muscle fibres, and are each controlled by sympathetic nerve fibres. As is true of the sympathetic nervous system in general, the path from the central nervous system to the periphery is made up of two sets of nerve fibres—the pre-ganglionic and the post-ganglionic fibres. The pre-ganglionic fibres, which control the sphincter iridis, arise from a definite group of cells (the Edinger-Westphal nucleus) in the anterior part of the nucleus of the third nerve and run to the ciliary ganglion. From this ganglion the post-ganglionic fibres arise and are distributed through the short ciliary nerve to the sphincter. The path controlling the dilator muscle is longer, its pre-ganglionic fibres arise from cells in the spinal cord which are probably situated in the lateral horn, about the level of the junction of its thoracic and cervical portion. The fibres leave the cord mostly by the first thoracic nerve, and run in the cervical sympathetic to the superior cervical ganglion. From this structure post-ganglionic fibres arise which, after a circuitous route, reach the eye and the dilator muscle of the iris through the long ciliary nerves. The afferent path of the reflex arc, which governs the action of the pupil to light, is also not absolutely determined. The impulses start in the retina, and it was at first assumed that the visual and reflex fibres were the same, and that the paths only separated beyond the primary optical centres, but later observations seem to indicate that the reflex fibres and the visual fibres are not identical. These primary centres are the lateral geniculate body, the anterior corpora quadrigemina and the pulvinar of the optic thalamus; and in man most of the fibres end in the geniculate body. According to Bernheimer, the pupillary fibres leave the optic tract just before they reach the external geniculate body, and run towards the middle line within the white matter of the corpora quadrigemina, where they turn, some going to the grey matter of this structure, and others directly to the region of the sphincter centres in the third nucleus. On the other hand, Bach was unable to confirm Bernheimer's results. Bach and Meyer also found that section of the cervical cord 1 mm. or 2 mm. below the medulla caused no loss of the pupillary reflex, but that if the cut passed just at the spinal end of the fourth ventricle, the reflex was abolished. If the cut were unilateral, it was the reflex of the opposite eye which was lost. They, therefore, suppose that in this region there is a centre of importance for the light reflex. Maxina finds that in cases of tabes and general paralysis, where the pupils have reacted normally during life the cells of the ciliary ganglion are intact; whereas when abnormal pupil reactions have occurred, there are more or less evident changes in the cells of this ganglion. The general result of work on the sympathetic system is, however, against the view that this ganglion can act as a reflex centre.

**Trifacial Neuralgia.**—In a clinical lecture on this subject Frazier[7] says that in the great majority of cases the cause is unknown, though exceptionally there may be a source of peripheral irritation. From a pathological point of view there are three separate and distinct types—one in which the lesion is a peripheral neuritis, the second in which it is in the Gasserian ganglion, and the third in which it is in the central nervous system. Unfortunately, it is not possible to determine clinically which cases are due to a peripheral neuritis and which are caused by disease of the ganglion. Any one, or two, or all three of the divisions of the nerve may be affected, and there are corresponding areas of pain and "tender points." It is rare for the pain to begin or to remain localised in the first division. The pain is spontaneous and paroxysmal, and there may be associated motor, vaso-motor, and trophic disturbances. In classifying cases it is of importance to distinguish the migrainous type, which generally occurs in women in early life suffering from migraine. After a number of years it becomes chronic and localised in one or more of the divisions of the trigeminus—in these cases the lesion is central and no operation will benefit. The other type, affecting men and women, occurs after the age of 40, and is due to changes either in the ganglion or its branches. As regards treatment, a certain proportion (? 20 per cent.) can be cured by medicines. Predisposing causes must be removed, and then strychnine administered hypodermically in heroic doses, beginning with $\frac{1}{30}$ grain daily and gradually increasing this to $\frac{1}{10}$ or $\frac{1}{8}$, or even higher. The patient should be kept in bed and under close observation. In cases of but one or two years' standing this will arrest or control the disease almost invariably. If this fails operative measures must be undertaken. If the pain be confined to one branch, *e.g.* the inferior dental or intraorbital, it may be hoped that a peripheral lesion is the cause, and the nerves should be divided at their entrance to the foramina with interposition of rubber tissue to prevent regeneration. This cures a certain percentage of cases, and produces a varying period of remission in the remainder, and is unattended with risk. When recurrence follows peripheral operations, or if the pain be more widespread, an intracranial operation is necessary. One of two things may be done—either extirpation of the Gasserian ganglion, or division of its sensory root proximal to the ganglion. Frazier advocates the latter, and reports three cases in which he had performed it with success. It has the advantage over extirpation of obviating a number of difficulties—being practically completed on exposing the ganglion, which is very troublesome to remove: the Hartley-Krause or temporal route is chosen, and the sensory root severed. It is found that pain does not return, owing to the fact that regeneration does not occur in sensory roots proximal to their root-ganglia. Frazier is inclined to practise temporary closure of one carotid artery as a means of controlling the troublesome hæmorrhage met with. This has been done in removal of a brain tumour, and excision of the superior maxilla, without impairing the functions of the brain, and might well be practised in these cases.

6 Amer. Jour. Med. Sc., Dec. 7 Ibid.

